# The Alcohol Dehydrogenase System in the Xylose-Fermenting Yeast *Candida maltosa*

**DOI:** 10.1371/journal.pone.0011752

**Published:** 2010-07-23

**Authors:** Yuping Lin, Peng He, Qinhong Wang, Dajun Lu, Zilong Li, Changsheng Wu, Ning Jiang

**Affiliations:** 1 Centre of Microbial Biotechnology, Institute of Microbiology, Chinese Academy of Sciences, Beijing, China; 2 Graduate School, Chinese Academy of Sciences, Beijing, China; 3 Tianjin Institute of Industrial Biotechnology, Chinese Academy of Sciences, Tianjin, China; Newcastle University, United Kingdom

## Abstract

**Background:**

The alcohol dehydrogenase (ADH) system plays a critical role in sugar metabolism involving in not only ethanol formation and consumption but also the general “cofactor balance” mechanism. *Candida maltosa* is able to ferment glucose as well as xylose to produce a significant amount of ethanol. Here we report the ADH system in *C. maltosa* composed of three microbial group I ADH genes (*CmADH1*, *CmADH2A* and *CmADH2B*), mainly focusing on its metabolic regulation and physiological function.

**Methodology/Principal Findings:**

Genetic analysis indicated that *CmADH2A* and *CmADH2B* tandemly located on the chromosome could be derived from tandem gene duplication. *In vitro* characterization of enzymatic properties revealed that all the three CmADHs had broad substrate specificities. Homo- and heterotetramers of CmADH1 and CmADH2A were demonstrated by zymogram analysis, and their expression profiles and physiological functions were different with respect to carbon sources and growth phases. Fermentation studies of ADH2A-deficient mutant showed that *CmADH2A* was directly related to NAD regeneration during xylose metabolism since CmADH2A deficiency resulted in a significant accumulation of glycerol.

**Conclusions/Significance:**

Our results revealed that *CmADH1* was responsible for ethanol formation during glucose metabolism, whereas *CmADH2A* was glucose-repressed and functioned to convert the accumulated ethanol to acetaldehyde. To our knowledge, this is the first demonstration of function separation and glucose repression of ADH genes in xylose-fermenting yeasts. On the other hand, *CmADH1* and *CmADH2A* were both involved in ethanol formation with NAD regeneration to maintain NADH/NAD ratio in favor of producing xylitol from xylose. In contrast, *CmADH2B* was expressed at a much lower level than the other two CmADH genes, and its function is to be further confirmed.

## Introduction

Alcohol dehydrogenase (ADH), which catalyzes the interconversion between acetaldehyde and ethanol, plays a central role in ethanol production and assimilation. Moreover, as NAD(H) or NADP(H) takes part in the reaction, ADH is involved in the general “cofactor balance” mechanism [1]. Yeast ADH belongs to the group I long chain (approximately 350 residues per subunit) zinc-dependent enzymes of microbial NAD- or NADP-dependent dehydrogenases [2]. Although the primary nucleotide and amino acid sequences of yeast ADHs are highly conserved, the members, physiological functions and metabolic regulations of the ADH systems vary among different yeast species. Furthermore, only one or two essential ADH genes are highly expressed and responsible for ethanol formation and assimilation in the majority of yeasts during glucose or xylose metabolism.

In *Saccharomyces cerevisiae*, *ScADH1* encodes the classical fermentative enzyme responsible for ethanol generation, and is expressed in large amounts in the presence of glucose [3], [4]. *ScADH2* encodes the enzyme that converts ethanol to acetaldehyde, and is negatively regulated by glucose [5]. Recently, Thomson *et al.* [6] resurrected the last common ancestor of ScADH1 and ScADH2 using ancestral sequence reconstruction and kinetic analysis, and identified that the ancestor was optimized in favor of making (not consuming) ethanol, resembling the modern ScADH1. After the ScADH1/ScADH2 duplication, ScADH2 conferred a novel function of consuming ethanol. In contrast to function separation and glucose-dependent regulation of *ADH1* and *ADH2* in *S. cerevisiae*, *ADH1* [7], [8] of *Pichia stipitis*, a natural xylose-fermenting yeast which is well studied for ethanol production, encodes the principal ADH with both fermentative and assimilatory functions, and is induced by oxygen limitation. *PsADH2* [8] is not expressed under aerobic or oxygen-limited conditions unless *PsADH1* is disrupted.

In xylose-fermenting yeasts, D-xylose is first reduced to xylitol and sequentially oxidized to D-xylulose by xylose reductase (XR) and xylitol dehydrogenase (XDH), respectively [9]. Cofactor imbalance would arise under anaerobic or oxygen-limited conditions since XDH is considered to be specific for NAD, while XR predominantly uses NADPH and no mechanism exists to reduce NADP with NADH [10]. In *P. stipitis*, the dual cofactor (NADPH and NADH) specificity of XR [11] could partially make up the cofactor imbalance and thus it could efficiently ferment xylose to ethanol under oxygen-limited conditions. While in some xylose-fermenting yeasts, such as *Candida tropicalis* and *Candida guilliermondii*, xylitol is largely accumulated due to the cofactor imbalance between NADPH-dependent XR and NAD-dependent XDH [12]. Our previous results [13] showed that *C. maltosa* accumulated xylitol with high substrate consumption rates and product yields in the batch fermentation under oxygen-limited conditions. XR of *C. maltosa* was exclusively NADPH-dependent, but NADP-dependent XDH activities were detected, which leaded to a significant accumulation of ethanol. Furthermore, *C. maltosa* showed a strong ability to produce ethanol from glucose similar to that of *S. cerevisiae* under aerobic conditions. However, the ADH system related to ethanol production of *C. maltosa* has not yet been studied in detail.

Hence, the objective of this study was to identify, characterize and elucidate composition and regulation of the ADH system in *C. maltosa* and its physiological function during glucose or xylose metabolism. As a consequence, the investigation would contribute to a better understanding of regulatory properties of fermenting both glucose and xylose to produce ethanol and other high-valued bio-products, e.g. xylitol, in natural xylose-utilizing yeasts.

## Results

### Cloning and genetic analysis of three distinct ADH genes in *C. maltosa*

Based on sequences of the ADH genes of *Candida albicans* [14] and *C. tropicalis* [15], two distinct DNA fragments harboring *C. maltosa* ADH genes were successfully obtained (Figure S1). One DNA fragment of 4415 bp was confirmed to contain a 1053-bp long uninterrupted open reading frame (ORF) showing high similarity with *C. albicans ADH1* (87.7%) and *C. tropicalis ADH1* (87.3%), and therefore this ORF was designated *CmADH1*. The other DNA fragment of 4660 bp was interestingly found to contain two tandem 1050-bp long ORFs that were both similar to *C. albicans ADH2*. Thus, the upstream and downstream ORFs were designated *CmADH2A* and *CmADH2B*, respectively (Figure S1). The phenomenon of tandem adjacent *CmADH2A* and *CmADH2B* was also confirmed in some other strains of *C. maltosa* (ATCC 28140 and AS 2.1386) by PCR cloning (Table S1) and subsequent sequencing.

The alignment of ADHs from different yeasts manifested that all the three *C. maltosa* ADH proteins seemed to be localized in the cytoplasm, because they did not possess N-terminal mitochondrial targeting signals (Box I, Figure S2) as described in *S. cerevisiae* ADH3 [16] and *Kluyveromyces lactis* ADH3 and ADH4 [17]. In addition, two typical motifs of the microbial group I ADHs [2] were found in CmADHs. One motif (Box II, Figure S2) matched the Zn-binding consensus (GHEXXGXXXXXGXXV). The other motif (Box III, Figure S2) was similar to the GXGXXG fingerprint pattern of the NAD-binding domain.

**Figure 1:**
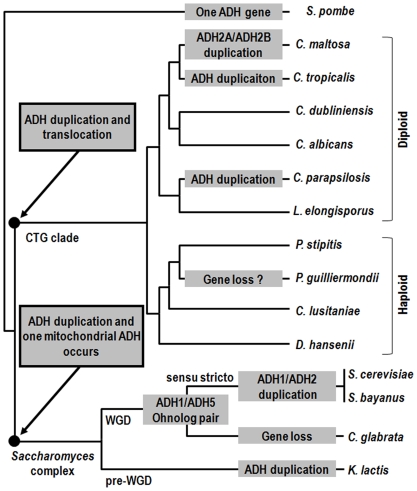
Minimum number of events required to explain evolution of ADH genes in some Saccharomycotina species. ADH duplication events are shown in gray boxes. The topology of the phylogenetic relationships was a composite drawn from several sources [15], [46], [47]. Major clades were named, including the *Saccharomyces* complex, the CTG clade containing species that translate codon CTG as serine instead of leucine, the group of species that share the whole-genome duplication (WGD) and the *Saccharomyces sensu stricto* group. The ADH gene duplication and gene loss events in the CTG clade were deduced based on comparative analysis of the genomic contexts of ADH homologs from species of this clade (Figure S3). The ADH duplication events in the *Saccharomyces* complex were reported previously [6], [18], and confirmed with the genomic contexts of ADH homologs from the Yeast Gene Order Browser (YGOB), an online tool for visualizing comparative genomics of yeasts [47]. ADH1/ADH5 ortholog pair was retained in *S. cerevisiae* and one copy has been lost in *Candida glabrata*. *K. lactis*, a pre-WGD yeast, has duplicated the ADH genes independently more than once after separating from the post-WGD yeast species.

To better understand the phenomenon of tandem adjacent *CmADH2A* and *CmADH2B* and evolution of the ADH system in *C. maltosa*, we deduced the process of gene duplication and gene loss of ADH homologs from some Saccharomycotina species. Based on those reported previously [6], [18], the evolutionary model of yeast ADH genes was enriched as follows (Figure 1). The ancestral yeast species contained only one cytoplasmic ADH. After divergence from *Schizosaccharomyces pombe*, the ADH in the ancestor of the *Saccharomyces* complex was duplicated and one copy became localized to the mitochondrion. In contrast, the ADH in the ancestor of the CTG clade was independently duplicated since there were no conserved gene orders or contents in *ADH* regions between the CTG clade and the *Saccharomyces* complex, and two duplicated ADH genes were retained to encode cytoplasmic ADHs. Furthermore, more ADH duplications occurred in some diploid species than in haploid species in the CTG clade. Comparative analysis of genomic contexts of ADH homologs from species of the CTG clade exhibited that *CmADH1* and *CmADH2A* were the orthologs of *PsADH1* and *PsADH2*, respectively (Figure S3). Following the speciation of diploid and haploid species in the CTG clade, *C. maltosa* has independently undergone once tandem ADH gene duplication event in its evolutionary history, resulting in modern tandem adjacent *CmADH2A* and *CmADH2B*. *C. tropicalis* and *Candida parapsilosis* seemed to have also duplicated their ADH gene(s) independently in their respective evolutionary history, resulting in their modern ADH systems encoded by more than two ADH genes, but none of their ADH genes were adjacent (genomic contexts of ADH genes other than *ADH1* and *ADH2* were not shown).

### *In vitro* characterization of recombinant *C. maltosa* ADH proteins

**Figure 2:**
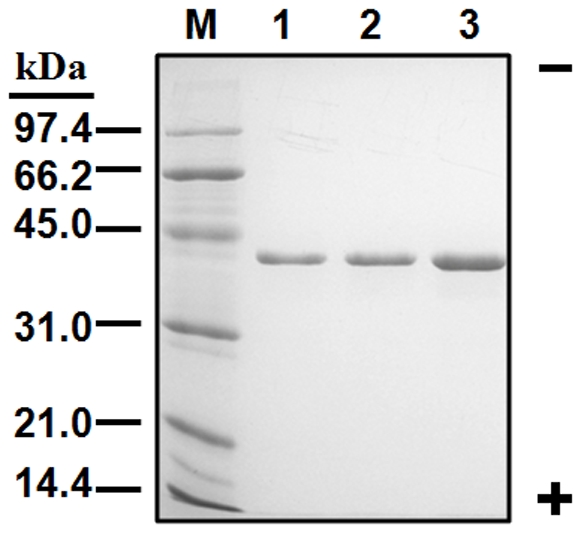
SDS-PAGE of purified recombinant CmADH1 (lane 1, 5 µg), CmADH2A (lane 2, 5 µg) and CmADH2B (lane 3, 10 µg). The standard proteins were applied to lane M.

The recombinant CmADH proteins with His-tags were purified and used for subsequent characterization of enzymatic properties as described in Materials and Methods. The clear single band of each recombinant CmADH corresponding to about 40 kDa protein was observed by SDS-PAGE (Figure 2).

ADH activities of CmADHs were tested with NAD or NADP as cofactor to determine cofactor preference. The specific activity of CmADH1, CmADH2A and CmADH2B with NAD was 24, 31 and 11 times higher than those with NADP (Table S2), respectively. These data indicated that all the three CmADHs preferred NAD to NADP as cofactor. The kinetic parameters of CmADHs for the substrates (ethanol and acetaldehyde) and the cofactors (NAD and NADH) were examined (Table 1). Among the three CmADHs, CmADH1 showed the lowest affinities to the cofactors, while CmADH2B showed the lowest affinities to the substrates. Moreover, all the three CmADHs showed more similar *K*_m(ethanol)_ values to those reported for ScADH1(17–24 mM) than for ScADH2 (0.6–0.8 mM) [6].

**Table 1:** Kinetic properties of CmADH1, CmADH2A and CmADH2B.

	*K*m (mM)		
Substrate	CmADH1	CmADH2A	CmADH2B
Ethanol	10.00±0.224	21.63±0.405	80.40±1.072
Acetaldehyde	0.161±0.018	0.155±0.012	22.58±0.638
NAD	1.21±0.095	0.288±0.016	0.317±0.044
NADH	1.53±0.183	0.051±0.004	0.031±0.002

Shown are mean and S.E. (*n* = 3).

**Figure 3:**
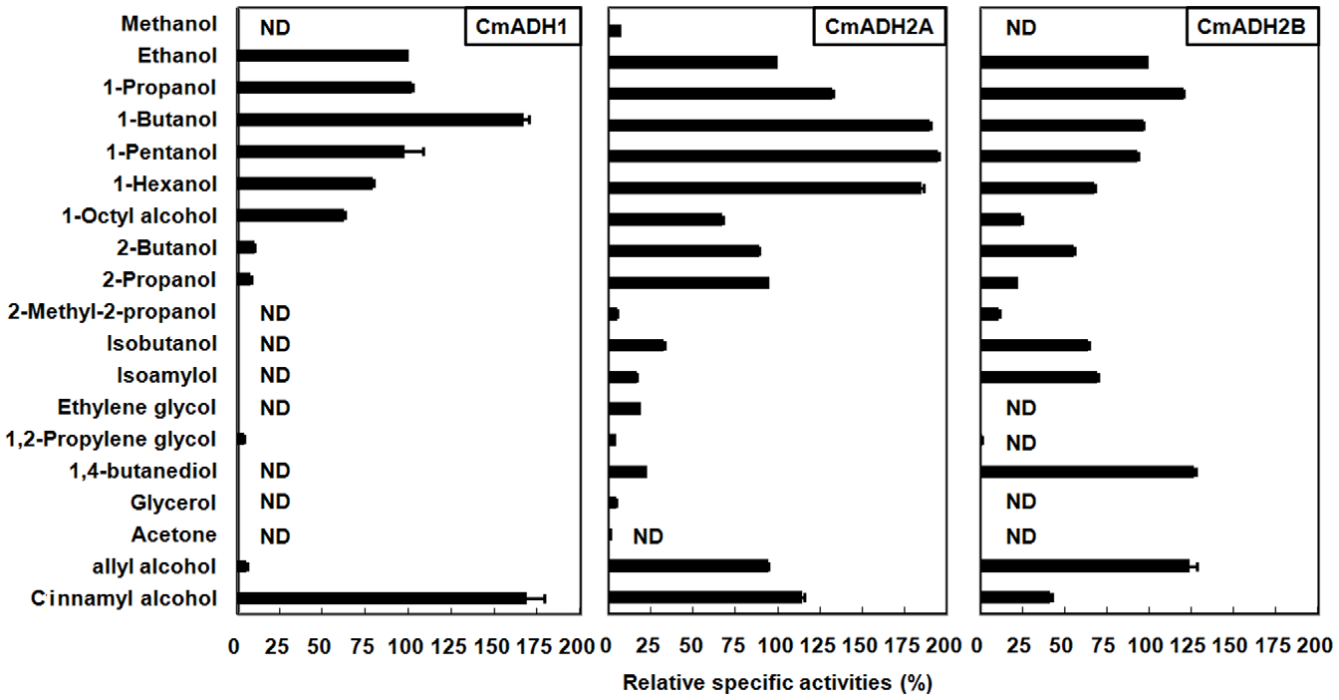
Relative specific activity of CmADH1, CmADH2A and CmADH2B on various alcohols. Relative specific activities of 100% corresponded to the specific activity on ethanol. The values were expressed as percent of the rate obtained with ethanol as substrates. Shown are mean ± S.E. (n = 3). *ND*, no detectable activity.

ADH activities of CmADHs towards nineteen alcohols, including aliphatic, aromatic and unsaturated alcohols, were measured to characterize their substrate specificities (Figure 3). All the three CmADHs showed a single peak in activity towards primary alcohols with C1–C7 carbon chains, although the carbon chain length of the primary alcohol which gave the highest relative activity was different. CmADH1 showed almost no activity towards secondary and branched alcohols. CmADH2A had higher relative activities towards secondary alcohols such as 2-propanol and 2-butanol than CmADH2B, while the relative activities of CmADH2B toward branched alcohols were higher than those of CmADH2A. CmADH1 and CmADH2A had low relative activities toward diols, but CmADH2B showed a very high relative activity on 1,4-butanediol. As for glycerol and acetone, all the three CmADHs had no or very low activities. Interestingly, CmADH2A and CmADH2B had high activities toward allyl alcohol that were identical to ethanol, but CmADH1 had a low relative activity of 6.04%. In addition, CmADH1 and CmADH2A showed higher relative activities toward cinnamyl alcohol than CmADH2B.

### Expression profiles and physiological functions of ADH genes in *C. maltosa* on different carbon sources and during sugar metabolism

**Figure 4:**
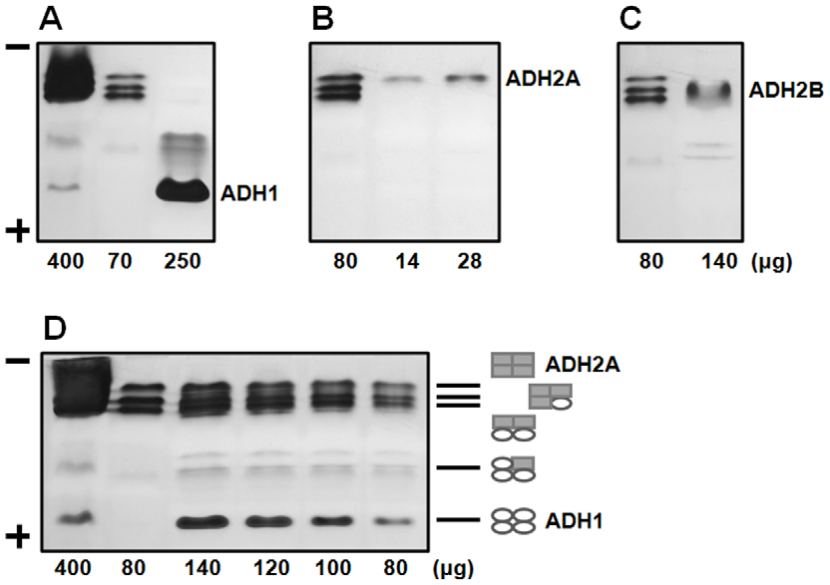
Zymogram analysis of ADH isozymes from *C. maltosa* Xu316 and recombinant *E. coli* BL21(DE3). Lane 1 in all figures and lane 2 in **A** and **D**: crude extracts from Xu316 cells grown for 18 h in YP medium containing 80 g/l xylose. Lane 3 in **A**, lane 2 and 3 in **B**, and lane 2 in **C**: crude extracts from *E. coli* BL21(DE3) overexpressing the recombinant CmADH1 (**A**), CmADH2A (**B**) and CmADH2B (**C**) without His-tags, respectively. **D.** Mixed crude extracts of the recombinant CmADH1 and CmADH2A. Amounts of proteins were given on the bottom of the lanes. Hypothetical isozyme composition of the major ADH bands in the electrophoretic pattern of *C. maltosa* was shown on the right of the lanes.

First of all, the correspondence between the native and recombinant CmADH isozymes was determined by zymogram analysis (Figure 4). *C. maltosa* produced multiple forms of ADH isozymes (Figure 4A, lane 1 and 2) including five clearly separated moving bands. The recombinant CmADH1 (Figure 4A, lane 3) and CmADH2A (Figure 4B, lane 2 and 3) comigrated with the fastest and the slowest moving band present in crude extracts of *C. maltosa*. The recombinant CmADH2B (Figure 4C, lane 2) displayed a more diffuse band that comigrated with another two slow-moving bands. Furthermore, the mixed crude extracts of the recombinant CmADH1 and CmADH2A (Figure 4D) were identified a similar electrophoretic pattern of ADH isozymes to crude extracts of *C. maltosa*, while other mixtures of crude extracts from recombinant *E. coli* (CmADH1 and CmADH2B, CmADH2A and CmADH2B) did not reveal the similar patterns (data not shown). Therefore, the fastest and the slowest moving isozymes could be the CmADH1 homotetramer and the CmADH2A homotetramer, respectively, and three middle migrating bands should represent heterotetramers formed between the *CmADH1* and *CmADH2A* gene products with different ratio (1∶3, 2∶2 and 361) (Figure 4D). Similar heterotetramer formation between ADH isozymes has also been reported in *S. cerevisiae* [19], [20] and *K. lactis* [21], [22]. In addition, there seemed to be no clear band corresponding to the ADH isozyme encoded by *CmADH2B* in crude extracts of *C. maltosa*. This has implicated that CmADH2B was probably produced at a very low level, or not produced at all under normal physiological conditions.

**Figure 5:**
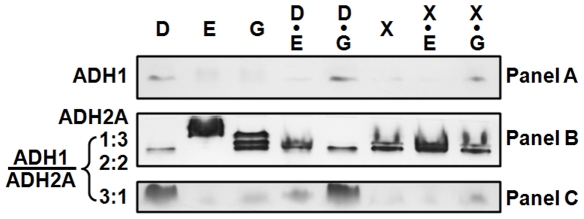
Expression of CmADH isozymes of *C. maltosa* Xu316 on different carbon sources. *C. maltosa* cells were grown in YP medium containing different carbon sources: 80 g/l glucose (D), 2% (V/V) ethanol (E), 2% (V/V) glycerol (G), 80 g/l glucose and 2% (V/V) ethanol (D•E), 80 g/l glucose and 2% (V/V) glycerol (D•G), 80 g/l xylose (X), 80 g/l xylose and 2% (V/V) ethanol (X•E), and 80 g/l xylose and 2% (V/V) glycerol (X•G). Crude extracts (110 µg protein) were prepared from cells in the mid-exponential phase (for 12 h) for zymogram analysis.

To examine the distinctive expression modes of *CmADHs*, protein profiles of CmADH isozymes were investigated with respect to carbon sources (Figure 5). When *C. maltosa* cells were grown in medium containing the fermentable carbon source glucose or xylose, CmADH isozymes were mainly composed of the CmADH1 homotetramer (Panel A) and the heterotetramers of CmADH1 and CmADH2A, while the CmADH2A homotetramer (Panel B, top band) was undetectable in the presence of glucose or faintly detectable in the presence of xylose. However, when *C. maltosa* cells were grown in the presence of ethanol as a sole carbon source (lane 2), the CmADH2A homotetramer was the only detectable CmADH isozyme. These results have implicated that *CmADH1* could be expressed during fermentative metabolism to reduce acetaldehyde to ethanol, while *CmADH2A* should be expressed under respiratory conditions to serve ethanol assimilation.

**Figure 6:**
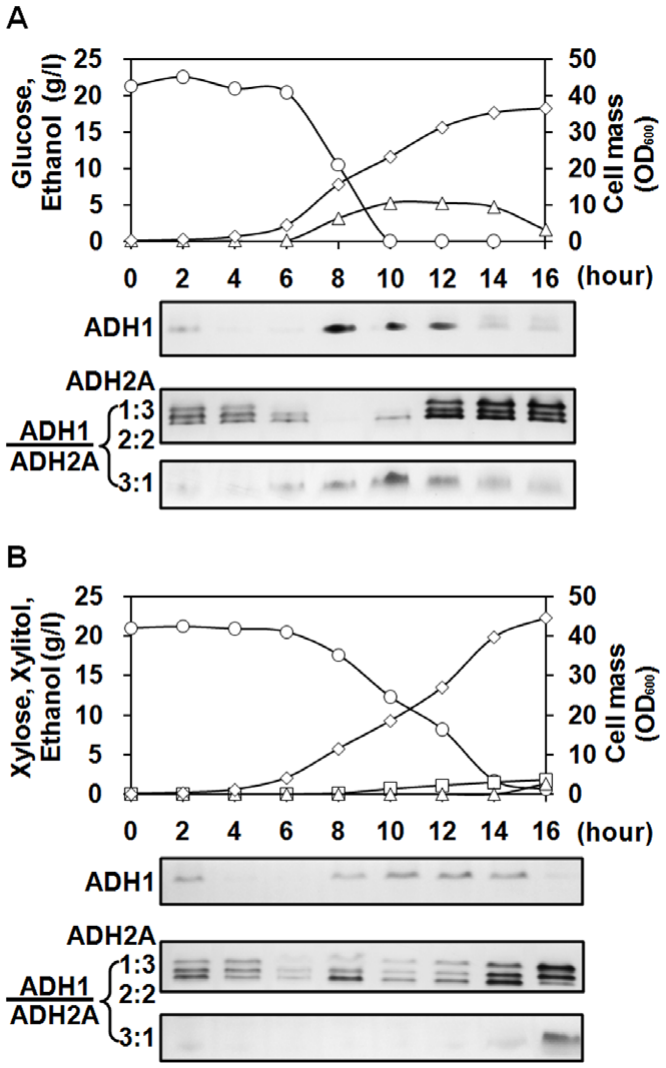
Expressions of CmADH isozymes of *C. maltosa* Xu316 during glucose (A) or xylose (B) metabolism under aerobic conditions. Glucose or xylose (*open cycles*) concentration, ethanol (*open triangles*) and xylitol (*open squares*) concentrations, and cell density (*open diamonds*) were determined at various times after inoculation. Crude extracts were prepared from cells at various times for zymogram analysis. Amounts of each gel were 100 µg or 10 µg.

To further elucidate the respective physiological functions of *CmADH1* and *CmADH2A* during sugar metabolism, expression profiles of CmADH isozymes were analyzed (Figure 6). During glucose metabolism, the CmADH1 homotetramer was gradually produced after the exponential phase initiated, whereas the CmADH2A homotetramer seemed to increase from the late exponential phase after glucose was consumed. At the same time, the heterotetramers of CmADH1 and CmADH2A as 1∶3 and 2∶2 increased with CmADH2A overproduction. These results suggested that *CmADH1* was responsible for ethanol formation, while *CmADH2A* was glucose-repressed and responsible for converting the accumulated ethanol to acetaldehyde after glucose was consumed. As for xylose metabolism, the expression profiles of CmADH isozymes were nearly similar to those during glucose metabolism. However, the expression of *CmADH2A* during xylose metabolism was initiated in the early exponential phase and much earlier than during glucose metabolism, which implicated that the physiological function of *CmADH2A* would be different during glucose and xylose metabolism.

### Screening and characterization of ADH2A-deficient mutants and physiological function of *CmADH2A*

*C. maltosa* ADH2A-deficient mutant was isolated and characterized to further reveal the physiological function of *CmADH2A* especially during xylose metabolism. The selection procedure was based on the ability of ADH to oxidize allyl alcohol to acrolein, an unsaturated aldehyde which is very toxic to cells [23]. When cells were incubated on media supplemented with a certain concentration of allyl alcohol, only those clones with reduced ADH activity were able to grow. It is known that allyl alcohol can be used as a select agent to isolate ADH-deficient mutants of some yeast species, such as *S. cerevisiae* [19], [24], *K. lactis* [25], [26] and *C. guilliermondii* [27]. Similarly, six mutants of *C. maltosa* Xu316, resistant to 400 mM allyl alcohol, were isolated. After zymogram analysis, the CmADH2A homotetramer dramatically decreased in these mutants (Figure S4), which indicated that all the mutants should be ADH2A-deficient. However, CmADH2A was still produced at a low level in the mutants because the heterotetramer of CmADH1 and CmADH2A as 3∶1 was detected. We chose one of the six mutants, M3-400, for subsequent investigations.

**Figure 7:**
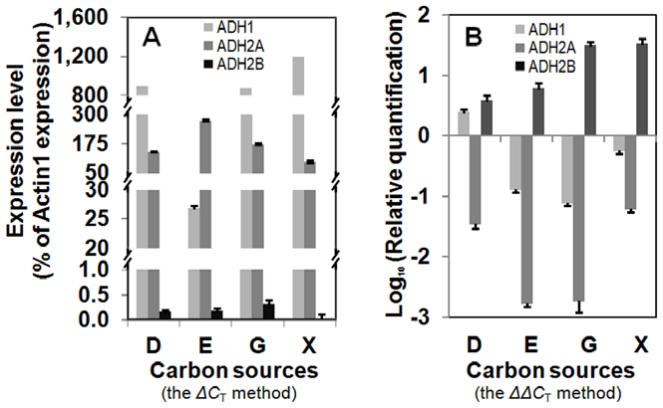
mRNA levels of three CmADH genes in wide type strain Xu316 (A) and their changes in ADH2A-deficient mutant M3-400 (B). *C. maltosa* cells were grown in YP medium containing different carbon sources under aerobic conditions: 80 g/l glucose (D), 2% (V/V) ethanol (E), 2% (V/V) glycerol (G), and 80 g/l xylose (X). Cells were harvested in the mid-exponential phase (for 12 h in glucose medium, 16 h in ethanol or glycerol medium and 24 h in xylose medium) for quantitative real-time RT-PCR.

To determine whether expression changes of *CmADH2A* occurred at the transcriptional level, mRNA levels of CmADHs in wild type strain Xu316 and ADH2A-deficient mutant M3-400 grown on different carbon sources under aerobic conditions were investigated and compared (Figure 7). In wild type strain (Figure 7A), *CmADH1* was significantly expressed in all substrates except for in ethanol, while *CmADH2A* was more highly expressed than *CmADH1* only in ethanol medium. The third ADH gene, *CmADH2B*, showed very low expressions in all substrates. These results were consistent to the previous zymogram analysis of CmADH isozymes (Figure 5). In ADH2A-deficient mutant, transcription of *CmADH2A* dramatically decreased (Figure 7B). CmADH2A deficiency slightly increased *CmADH1* expression in glucose medium and decreased *CmADH1* expression in other substrates. On the other hand, CmADH2A deficiency significantly increased *CmADH2B* expression from 3 fold to 33 fold in either fermentable or non-fermentable carbon source especially xylose and glycerol, but *CmADH2B* expression was still much lower than *CmADH1* expression in ADH2A-deficient mutant. Therefore, mutations of CmADH2A deficiency might occur at the transcriptional level rather than in the structural gene since *CmADH2A* was still transcribed and expressed at a very low level in ADH2A-deficient mutant, and CmADH2A deficiency had different effects on the transcriptions of *CmADH1* and *CmADH2B*.

ADH2A-deficient mutant M3-400 was compared to wild type strain Xu316 for the same sugar fermentation (glucose or xylose) under the same aeration conditions (aerobic or oxygen-limited) (Table 2). Compared with Xu316, the maximum substrate consumption rates (q_s_max) decreased by approximately half in ADH2A-deficient mutant M3-400, while CmADH2A deficiency had little effect on the maximum specific growth rates (μ_max_) or the biomass yields (Y**_x/s_**). At the same time, CmADH2A deficiency had almost no effect on the ethanol yields (Y_p/s_, ethanol) from glucose, but resulted in lower ethanol yields and significant accumulations of glycerol from xylose. Intriguingly, the xylitol yield (Y_p/s_, xylitol) clearly decreased in ADH2A-deficient mutant M3-400 under oxygen-limited conditions. Furthermore, M3-400 accumulated more glycerol under oxygen-limited conditions than under aerobic conditions. Glycerol has been reported to serve as a redox sink by oxidizing the excess NADH to NAD in *S. cerevisiae* [28]. These results have confirmed that *CmADH2A* would facilitate ethanol production from xylose and seemed to be important for xylitol production of *C. maltosa* under oxygen-limited conditions.

**Table 2:** Comparison of fermentative parameters of wild type strain (Xu316) and ADH2A-deficient mutant (M3-400) of *C. maltosa*.

Sugars	Aeration	Strain	μ_max_ (h^−1^)	q_s_max g (g h)^−1^	Y_x/s_ (g g^−1^)	Y_p/s_, ethanol (g g^−1^)	Y_p/s_, xylitol (g g^−1^)	Y_p/s_, glycerol (g g^−1^)
Glucose	Aerobic	Xu316	0.66	0.80	0.28	0.37	NA	ND
		M3-400	0.69	0.39	0.30	0.34	NA	ND
	Oxygen limited	Xu316	0.47	0.86	0.08	0.44	NA	ND
		M3-400	0.42	0.49	0.08	0.44	NA	ND
Xylose	Aerobic	Xu316	0.25	0.11	0.39	0.14	0.32	0.00
		M3-400	0.22	0.09	0.39	0.05	0.33	0.06
	Oxygen limited	Xu316	0.10	0.24	0.29	0.11	0.39	0.00
		M3-400	0.09	0.14	0.26	0.05	0.26	0.11

NA, not applicable; ND, not detected.

## Discussion

*C. maltosa* shows great potential to utilize xylose [13], which is abundant in the renewable lignocellulosic biomass and one of important substrates for the future biotechnology applications. The ADH system is indispensable for sugar metabolism and ethanol production. However, the ADH system of *C. maltosa* has not yet been determined. Here we have cloned and sequenced three distinct structural ADH genes (*CmADH1*, *CmADH2A* and *CmADH2B*) from *C. maltosa*. Intriguingly, *CmADH2A* and *CmADH2B* were tandem adjacent genes, and this is the first reported phenomenon in yeast, although the ADH genes are often arranged in tandem in some other organisms, such as human and mouse [29]. Nucleotide and amino acid sequence analysis suggested that all the three CmADHs might be localized in the cytoplasm and fitted in the microbial group I ADHs.

*In vitro* characterization of enzymatic properties showed that all the three CmADHs were NAD-dependent, and had broad substrate specificities similar to ScADH2 [18], ADHs of *K. lactis* [30], *Candida utilis* ADH1 [31] and ADHs of *C. guilliermondii* [27]. But ScADH1 [18] is different from other yeast ADHs in its inability to catalyze longer chain alcohols. The narrow substrate specificity of ScADH1 has been reported to be the result of alterations in its substrate binding cleft that Met-270 of ScADH1 has substituted by Leu in other yeast ADHs [32]. Compared with CmADH1 and CmADH2A, CmADH2B had more different residues (Figure S2, indicated by reversed letters) in the substrate-binding pocket, which might be one of the factors contributing to the higher relative activities of CmADH2B toward some branched alcohols and 1,4-butanediol than CmADH1 and CmADH2A.

During glucose metabolism, *CmADH1* was induced in presence of glucose, while *CmADH2A* was glucose-repressed and largely expressed after glucose was consumed (Figure 5 and 6A). This means that *C. maltosa* converts glucose into ethanol via acetaldehyde with CmADH1, and then consumes the accumulated ethanol with CmADH2A. Moreover, CmADH2A seemed not to be responsible for ethanol production from glucose, which was confirmed by the fermentation studies of ADH2A-deficient mutant, where CmADH2A deficiency almost did not affect the ethanol yields from glucose (Table 2). Hence, the expression regulation and the physiological function of the CmADH1/CmADH2A system in *C. maltosa* were essentially similar to those of the ScADH1/ScADH2 system in *S. cerevisiae* during glucose metabolism [6], [33]. In contrast, the ancestral yeast species seems to contain one cytoplasmic ADH [18], and a dual function of an ADH gene responsible for both formation and consumption of ethanol have been described for *PsADH1* of *P. stipitis* [7], *PaADH1* of *Pichia anomala* [34] and *CaADH1* of *C. albicans* [14]. Gene duplications lead to the modern ADH system composed of more than one ADH genes during yeast evolution (Figure 1). Moreover, duplicated ADH genes have evolved to play different physiological functions and be regulated in different modes. The ScADH1/ScADH2 duplication, along with their function separation and glucose repression of ScADH2, provide the molecular basis for *S. cerevisiae* to have a remarkable trait of producing ethanol in high concentrations even in the presence of oxygen [6], [33]. Thus, the CmADH1/CmADH2A system would also enable *C. maltosa* to accumulate a large amount of ethanol from glucose. According to our fermentation studies, *C. maltosa* was truly able to ferment a high concentration of glucose to produce a large amount of ethanol even in presence of oxygen (data not shown).

Based on the expression analysis of CmADH isozymes and fermentation studies of ADH2A-deficient mutant, it was found that *CmADH1* and *CmADH2A* were both expressed and involved into ethanol formation during xylose metabolism. This has implicated that the ADH system of *C. maltosa*, a xylitol-producing yeast, is different from that of the ethanol-producing yeast *P. stipitis*, in which *PsADH1* is the critical gene responsible for ethanol production [7]. As for other xylitol-producing yeasts, no information is currently available regarding expression regulation and physiological functions of their ADH genes during xylose metabolism. It has been reported that oxygen limitation stimulates xylitol accumulation due to the inhibition of XDH activity by increased NADH concentration, and NAD regeneration is equally important for xylitol accumulation under oxygen-limited conditions [12]. *C. tropicalis* and *C. guilliermondii* can regenerate NAD by accumulation of ethanol and glycerol and by glycerol accumulation [12], respectively. CmADH2A deficiency partially reduced NAD regeneration due to the decreased ethanol yield whether the cells were grown under aerobic conditions or oxygen-limited conditions. Under aerobic conditions, NAD might be still efficiently regenerated via respiratory pathways and by glycerol accumulation, and thus the xylitol yield was not affected by CmADH2A deficiency. However, under oxygen-limited conditions, NAD regeneration via respiratory pathways is restricted, and ADH2A-deficient mutant seemed to regenerate NAD by accumulating more glycerol at the expense of the decreased xylitol yield. Therefore, the main physiological function of the ADH system in *C. maltosa* seemed to regenerate NAD by ethanol accumulation to maintain NADH/NAD ratio in favor of producing xylitol from xylose.

In contrast to *CmADH1* and *CmADH2A*, *CmADH2B* was expressed at a very low level in wild type strain and ADH2A-deficient mutant, although the expression was up-regulated to some extent with CmADH2A deficiency. In other yeasts, there are some similar ADH genes, e.g. *ADH5* in *S. cerevisiae*, which are not expressed under normal physiological conditions. However, ScADH5 can produce ethanol in an *ADH1*/*ADH3* double null mutant [35], and its transcription is up-regulated in a recombinant *S. cerevisiae* strain capable of anaerobic growth on xylose, which is not normally utilized by yeasts in the absence of oxygen [36]. *CmADH2B* was significantly up-regulated in ADH2A-deficient mutant grown on xylose, but its precise function remains to be investigated.

In summary, three microbial group I ADH genes of *C. maltosa*, *CmADH1*, *CmADH2A* and *CmADH2B*, were cloned and characterized. *CmADH1* had the similar physiological function to *ADH1* in *S. cerevisiae* and *P. stipitis*. However, the expression regulation and the physiological function of *CmADH2A* were more similar to *ScADH2* than to *PsADH2*, although *CmADH2A* were the ortholog of *PsADH2* according to comparative analysis of genomic contexts of ADH homologs. Furthermore, CmADH2A did not show a significantly low *K*_m_ for ethanol like ScADH2 [6], suggesting that the evolution of *CmADH2A* might be due to the acquirement of glucose repression instead of high affinity to ethanol. In terms of xylose metabolism in natural xylose-fermenting yeasts, previous studies mainly focus on metabolic reconstruction and optimization of xylose to xylulose-5-phosphate, which are catalyzed by XR, XDH and xylulokinase. We are now investigating regulation factors of CmADH2A to reveal the mechanism of glucose repression in *C. maltosa*. From an evolutionary standpoint, glucose repression of CmADH2A is presumably acquired after the speciation of *C. maltosa* and *P. stipitis*. Moreover, the regulation mechanism of glucose repression in *C. maltosa* might be different from that in *S. cerevisiae*, which is thought to basically derive from the whole genome duplication event [37]. All these information suggested that *C. maltosa* should be a potential model for studying yeast sugar metabolism, and a significant candidate for the future utilization of glucose and xylose from renewable lignocelluloses to produce fuel ethanol and other high-valued bio-products.

## Materials and Methods

### Strains, culture conditions and metabolite analysis

*C. maltosa* Xu316, a wild type strain from our laboratory collection, was used as a source for the ADH genes. *C. maltosa* ATCC 28140 and AS 2.1386 from China General Microbiological Culture Collection Center (CGMCC) were used as reference strains. *C. maltosa* M3-400, a spontaneous mutant deficient in ADH2A, was derived from Xu316 and isolated by using allyl alcohol as a select agent as reported for *C. guilliermondii* [27]. *E. coli* DH5α was used for cloning, and BL21(DE3) for overproduction of the recombinant *C. maltosa* ADH proteins.

Yeast cells were grown at 30°C in YP (per liter, 10 g yeast extract, 20 g peptone, pH 5.5) medium containing different carbon sources for different times as specified in the text, and collected for ADH expression analysis using zymogram analysis or quantitative real-time RT-PCR. Inocula were grown in flask for 12 h, and initial cells were adjusted to OD_600_ = 0.2. In non-controlled shake flask fermentation, aerobic and oxygen-limited conditions were achieved by varying the volume of medium and the speed of shaking as described earlier [38], [39] with YP medium containing 80 g/l glucose or xylose. Fermentation broth was centrifuged at 12000 r/min for 10 min and the supernatant was used to quantify metabolites. Glucose, xylose, xylitol, ethanol and glycerol were determined by HPLC with a refractive index (RI) detector and an Aminex HPX-87H column (Bio Rad Laboratories). Cell growth was monitored by measuring the turbidity at 600 nm. One OD unit corresponds to a dry weight of 0.429 g/l.

### Cloning and genetic analysis of *C. maltosa* ADH genes

The complete fragments of *C. maltosa* ADH genes were obtained by degenerate PCR and cassette-mediated PCR following with nested PCR (Figure S1). PCR reaction was performed using ExTaq DNA polymerase (TaKaRa) with genomic DNA of *C. maltosa* Xu316 as the template. Assembly of the sequenced PCR fragments and pairwise sequence comparison were performed using the computer software DNAMAN. Multiple sequence alignment was constructed with the Clustal×program [40]. Gene duplication events of yeast ADHs were studied using comparative analysis of their genomic contexts. The genomic contexts of yeast ADH genes used in this study were obtained from the following databases: the *Candida* database at the Broad Institute (*Candida* species and *Debaryomyces hansenii*), the GeneDB (*Candida dubliniensis*), the Map View of NCBI (*P. stipitis* and *P. guilliermondii*), and the Yeast Gene Order Browser (some yeast species in the *Saccharomyces* complex).

### Heterologous expression of recombinant CmADHs

The ORF fragments of *CmADH1*, *CmADH2A* and *CmADH2B* were subcloned into the *Nde*I and *Xho*I sites of the pET28a(+) vector or the pET21a(+) vector (Invitrogen), respectively (Table S1). All the plasmids containing CmADH genes were sequenced to verify sequence integrity (AuGCT, Beijing, China). The recombinant CmADH proteins overexpressed using the pET28a(+) vector were N-terminally His-tagged, and purified by affinity chromatography using a 5-ml nickel-charged HiTrap column (Pharmacia) according to the manufacturer's recommendations. The purified proteins were concentrated to 5–8 mg/ml by ultrafiltration using Amicon Ultra-4 centrifuge filter unit (30 kDa cut-off, Millipore), characterized by SDS-PAGE and used for *in vitro* characterization of enzymatic properties. On the other hand, the recombinant CmADH proteins overexpressed using the pET21a(+) vector were not His-tagged, and cell extracts of the corresponding *E. coli* BL21(DE3) transformant were used for zymogram analysis. Protein concentrations were determined by the method of Bradford [41].

### *In vitro* characterization of enzymatic properties of recombinant CmADHs

ADH activity was assayed as previously described [7] with a slight modification. The reaction mixture contained 100 mM Tris-HCl buffer (pH 8.3), 5 mM NAD, enzyme solution (1 to 50 µg of protein), and 100 mM ethanol, in a total volume of 1 ml. To determine cofactor preference, NADP (5 mM) was tested in ADH activity assays. One unit of enzyme activity was defined as the amount to reduce 1 µmol of NAD or NADP per minute at 25°C. Specific ADH activity (U/mg) was expressed as units (U) per mg of protein. To measure kinetics of CmADHs with ethanol, acetaldehyde, NAD and NADH, the experiments were performed as previously described [42] except for using sodium phosphate buffer (100 mM, pH 7.5). The experimental data were analyzed using Enzyme Kinetics Module of SigmaPlot 2001 (Systat Software Inc.) to get affinity constant (*K*_m_) values. To determine the substrate specificities of CmADHs towards a variety of alcohol substrates, ADH activity was assayed in the oxidative direction in the presence of the alcohol substrate (at 100 mM) with NAD as cofactor.

### Zymogram analysis

ADH activity was visualized by native PAGE [43] followed by active staining [27]. Crude extracts of *C. maltosa* were prepared by vortexing with glass beads as described previously [13] except for using 20 mM Tris-HCl (pH 7.9) as the extraction buffer. Proteins were separated on a 3% (wt/vol) stacking gel and 10% (wt/vol) separating gel with a constant voltage of 100 V for 160 min at 4°C. The electrophoresis gels were then stained as previously described [27].

### Quantitative real-time RT-PCR

Real-time PCR primers (Table S1) were designed based on the sequences around 500–600 bp of CmADH genes where they have high sequence diversity to confirm specific gene product formation. Actin1 gene was chosen as endogenous gene [44]. The amplicon lengths were all about 120 bp. Total RNA were extracted using TRIZOL® Reagent (Invitrogen). To avoid DNA contamination that would disturb the quantitative mRNA analysis, the RNA samples were treated with DNase I (TaKaRa) following the instructions of the kit. cDNA was synthesized using SuperScript™ III First Strand Synthesis System (Invitrogen) for real-time PCR. Real-time PCR was carried out with the ABI Prism® 7300 Sequence Detection System and the SYBR Green I fluorescent dye for DNA detection using SYBR Green PCR Master Mix (Applied Biosystems). The data were analyzed using the 2^−*Δ*CT^ and the 2^−*ΔΔ*CT^ method [45].

## Supporting Information

Table S1 Primers used in this study. *Nde* I and *Xho* I restriction sites are underlined. *Primer set ADH2Aup/ADH2Bdn was used to clone the DNA fragment containing *CmADH2A* and *CmADH2B* from the reference strains *C. maltosa* ATCC 28140 and AS 2.1386.
(0.06 MB DOC)

Table S2 Cofactor preference of CmADH1, CmADH2A and CmADH2B. Shown are mean and S.E. (n = 3).
(0.04 MB DOC)

Figure S1 The cloning strategy of *C. maltosa* ADH genes. Firstly, a 2565-bp DNA fragment containing the upstream sequence and the 5′ coding region of *CmADH1* and a 737-bp DNA fragment harboring partial sequence of *CmADH2* were amplified with degenerate primers ADH1-3/ADH1-4 and P1/P2 (Table S1) and sequenced (AuGCT, China), respectively. ADH1-3 was designed based on the sequence of *GPI10* gene upstream *ADH1* both in *C. albicans* and *C. tropicalis*, which encodes an integral membrane protein involved in glycosylphosphatidylinositol (GPI) anchor synthesis. And ADH1-4 was prepared in highly conserved regions of *C. albicans ADH1* and *C. tropicalis ADH1*. PCR primers P1 and P2 were designed according to *C. albicans ADH2*. Then, the remaining upstream and downstream sequences were obtained through several cassette-mediated PCRs using TaKaRa LA PCR™ *in vitro* Cloning Kit (TaKaRa), a PCR-based DNA walking method. The restriction endonucleases and genome-specific primers (Table S1) used were indicated on the top of each DNA fragment. Finally, the overlapping DNA fragments were assembled into the complete DNA fragment. The sequences of the coding regions and the 5′ and 3′ flanking regions of *CmADH1* and *CmADH2A-CmADH2B* have been deposited in the GenBank database under accession numbers GU395490 and GU395491, respectively.
(5.06 MB TIF)

Figure S2 Alignment of the deduced amino acid sequences of CmADHs with those of other yeast ADHs. Numbering of amino acid corresponds to ScADH1. Residues that are involved in catalyses, are headed by letters: a, adenine binding pocket; r, adenosine ribose binding; p, pyrophosphate binding; n, binding of nicotinamide or nicotinamide ribose; s, substrate binding pocket; b, poton relay system; z, ligands of the active site zinc atom or ligands of the structural zinc atom. m, five more strictly conserved residues among the microbial ADHs. Asterisks indicate conserved amino acid residues, eight glycine residues and one valine of ADHs from divergent sources. Box I, mitochondrial targeting region; Box II, zinc-binding consensus; Box III, NAD(P)-binding motif. Reversed letters indicate the residues involved in cofactor binding, substrate binding or catalysis which are not conserved in three CmADHs and ScADH1. Accession numbers (from CaADH1 to SpADH1): X81694, XM_712556, AF008245, AF008244, V01292, Z49212, AY692988, XM_456023, X64397, X62766, X62767, AL032681.
(2.49 MB TIF)

Figure S3 Genomic contexts of *ADH1* (A) or *ADH2* (B) homologs from species of the CTG clade are drawn according to their genomic sequences. Species names and gene identifiers are shown in each box. Orthologous gene boxes are represented in the same color. Arrows indicate directions of gene transcription and are not to scale. Connectors join nearby genes: a solid bar for adjacent genes, two gray bars for loci less than five genes apart and one gray bar for loci <20 genes apart. Genomic contexts in square brackets are conversed compared with *C. maltosa*. The species of the CTG clade exhibited highly conserved gene order around ADH1. In case of *ADH2* region, *C. maltosa* had the same genomic context as *C. tropicalis* except that another *ADH* existed in *C. maltosa*, and the other species of the CTG clade had transketolase gene *TKL1* instead of squalene epoxidase gene *ERG1*. In general, two ADH genes, *ADH1* and *ADH2*, existed in all the species of the CTG clade except for *Pichia* (*Candida*) *guilliermondii* in this study, although the *ADH* loci were dispersed in *Candida parapsilosis* and *Lodderomyces elongisporus*.
(1.34 MB TIF)

Figure S4 Characterization of ADH-deficient strain of *C. maltosa* by zymogram analysis. Cells were grown in YP medium containing 20 g/l glucose and harvested in the mid-exponential phase for zymogram analysis. Lane 1, wild-type strain Xu316. Lane 2–7, ADH-deficient mutants M3-360, M3-400, M1-400, M11-400, M12-400 and M15-400.
(0.22 MB TIF)
