# Supplementary material for: The Alcohol Dehydrogenase System in the Xylose-Fermenting Yeast Candida maltosa
Source: PLoS One. 2010 Jul 23;5(7):e11752. doi: 10.1371/journal.pone.0011752 (PMC2909261; doi:10.1371/journal.pone.0011752)
Supplement: Table S1 — Primers used in this study. Nde I and Xho I restriction sites are underlined. *Primer set ADH2Aup/ADH2Bdn was used to clone the DNA fragment containing CmADH2A and CmADH2B from the reference strains C. maltosa ATCC 28140 and AS 2.1386. (0.06 MB DOC) [file pone.0011752.s001.doc]

**Table S1.** Primers used in this study

| **Name** | **Sequence** |
| --- | --- |
| Gene cloning primers | |
| ADH1-3 | 5'-RTGGTTCTTGRCKTGTGAAC-3' |
| ADH1-4 | 5'-TYTTACCTTCTTCCATCAAT-3' |
| S11 | 5'-GCCATTGACCAATCTGTTGAATATGTTAGACC-3' |
| S12 | 5'-AACAGACAAGATACCGCTGAAGCCGTTGA-3' |
| P1 | 5'-GGCTGGAAAGTTGGTGATT-3' |
| P2 | 5'-GGTAATTCGGATAAGCCAAC-3' |
| S1 | 5'-AAAGCCTTGTAAACGGTAACACCAGCACAC-3' |
| S2 | 5'-GACAAGTCAGCTTGAGCACAGTTTGGTTCA-3' |
| S3 | 5'-GTGTTACCGTTTACAAGGCTTTGAAGACTGC-3' |
| S4 | 5'-GTCACTGCTCCAGTCTTTGACTCCGTCGTT-3' |
| S5 | 5'-AATCGGTGACTATGCTGGTATCAAATGGTT-3' |
| S6 | 5'-ATGATGGTACTTTCCAACAATACGCCACGG-3' |
| S7 | 5'-AAGTTTGTCAAATCATTAGGGGCGGAAGAA-3' |
| S8 | 5'-TGCTGAAGCTATTGATTTCTTTACCAGGGGTTT-3' |
| S9 | 5'-CCGCTTTCTTCGCAGTTTACTTTCTGTTTA-3' |
| S10 | 5'-CAGCGATAAAGCGGAGTAAAAGAAAGAAAGAAAG-3' |
| ADH2Aup* | 5'-TGAGTGGTCATCCTTTGCTA-3' |
| ADH2Bdn* | 5'-GGATTCCCACTTGCTTTACT-3' |
| Gene expression Primers | |
| ADH1e1 | 5'-GGAATTCCATATGTCTGAACAAATCCCAAAAACTC-3' |
| ADH1e4 | 5'-CCGCTCGAGTTATTTAGAGGTGTCCAAAACG-3' |
| ADH2Ae1 | 5'-GGAATTCCATATGTCTTCTATTCCAACTACTCAAAAAGC-3' |
| ADH2Ae4 | 5'-CCGCTCGAGTTATTTGGAGGTGTCCAAAACG-3' |
| ADH2Be1 | 5'-GGAATTCCATATGTATTCAATTCCAACTACCCAAAAAG-3' |
| ADH2Be4 | 5'-CCGCTCGAGTTACTTGGATGAGTCGACAACG-3' |
| Real time PCR primers | |
| CmACT-1s | 5'-TGGATGTCAGAAAAGAATTATACGGT-3' |
| CmACT-1a | 5'-CATAGAAGATGGAGCCAAAGCAGT-3' |
| CmADH1-1s | 5'-TGAAAACTGCTGACTTATCTGCC-3' |
| CmADH1-1a | 5'-CACCGTCAATACCAACAACTCTC-3' |
| CmADH2A-1s | 5'-GCTTTGAAGACTGCCAACTTACA-3' |
| CmADH2A-1a | 5'-CGTCAATGGCCAAGACTCTG-3' |
| CmADH2B-1s | 5'-TTTGATAACTGCAGAATTAATGCCT-3' |
| CmADH2B-1a | 5'-ATCAATCGCCAAAACCCGA-3' |

*Nde*I and *Xho*I sites are underlined. *Primer set ADH2Aup/ADH2Bdn was used to clone the DNA fragment containing *CmADH2A* and *CmADH2B* from the reference strains *C. maltosa* ATCC 28140 and AS 2.1386.
