# Supplementary material for: The Alcohol Dehydrogenase System in the Xylose-Fermenting Yeast Candida maltosa
Source: PLoS One. 2010 Jul 23;5(7):e11752. doi: 10.1371/journal.pone.0011752 (PMC2909261; doi:10.1371/journal.pone.0011752)
Supplement: Table S2 — Cofactor preference of CmADH1, CmADH2A and CmADH2B. Shown are mean and S.E. (n = 3). (0.04 MB DOC) [file pone.0011752.s002.doc]

**Table S2.** Cofactor preference of CmADH1, CmADH2A and CmADH2B

|  | **Specific activities (U/mg)** | |
| --- | --- | --- |
| **Isozyme** | **NAD** | **NADP** |
| CmADH1 | 1.03±0.34 | 0.04±0.42 |
| CmADH2A | 3.14±0.22 | 0.10±0.31 |
| CmADH2B | 3.64±0.33 | 0.32±0.27 |

Shown are mean and S.E. (*n* = 3).
