# Supplementary figures and images for: The Alcohol Dehydrogenase System in the Xylose-Fermenting Yeast Candida maltosa
Source: PLoS One. 2010 Jul 23;5(7):e11752. doi: 10.1371/journal.pone.0011752 (PMC2909261; doi:10.1371/journal.pone.0011752)

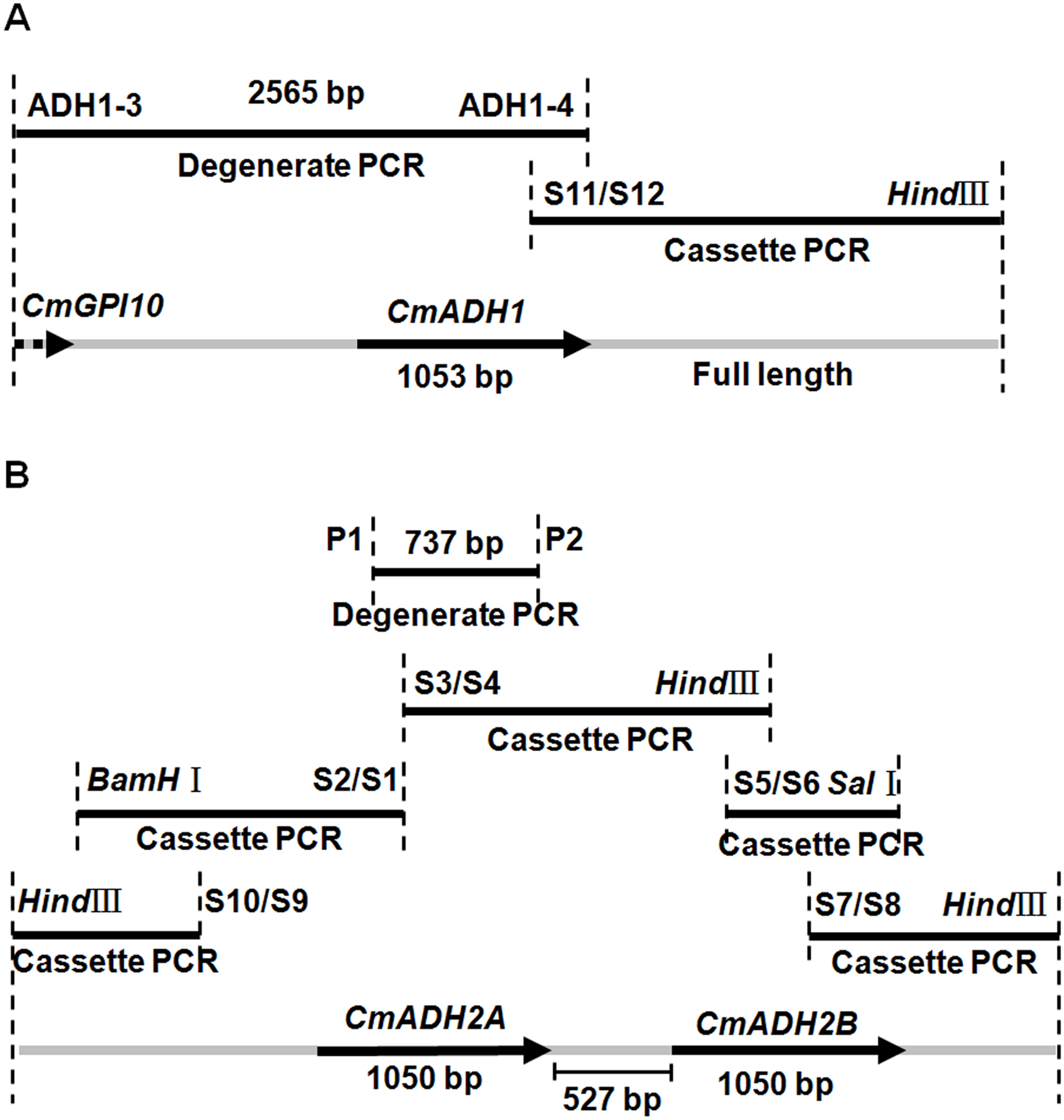

Supplement: Figure S1 — The cloning strategy of C. maltosa ADH genes. Firstly, a 2565-bp DNA fragment containing the upstream sequence and the 5′ coding region of CmADH1 and a 737-bp DNA fragment harboring partial sequence of CmADH2 were amplified with degenerate primers ADH1-3/ADH1-4 and P1/P2 (Table S1) and sequenced (AuGCT, China), respectively. ADH1-3 was designed based on the sequence of GPI10 gene upstream ADH1 both in C. albicans and C. tropicalis, which encodes an integral membrane protein involved in glycosylphosphatidylinositol (GPI) anchor synthesis. And ADH1-4 was prepared in highly conserved regions of C. albicans ADH1 and C. tropicalis ADH1. PCR primers P1 and P2 were designed according to C. albicans ADH2. Then, the remaining upstream and downstream sequences were obtained through several cassette-mediated PCRs using TaKaRa LA PCR™ in vitro Cloning Kit (TaKaRa), a PCR-based DNA walking method. The restriction endonucleases and genome-specific primers (Table S1) used were indicated on the top of each DNA fragment. Finally, the overlapping DNA fragments were assembled into the complete DNA fragment. The sequences of the coding regions and the 5′ and 3′ flanking regions of CmADH1 and CmADH2A-CmADH2B have been deposited in the GenBank database under accession numbers GU395490 and GU395491, respectively. (5.06 MB TIF) [file pone.0011752.s003.tif]

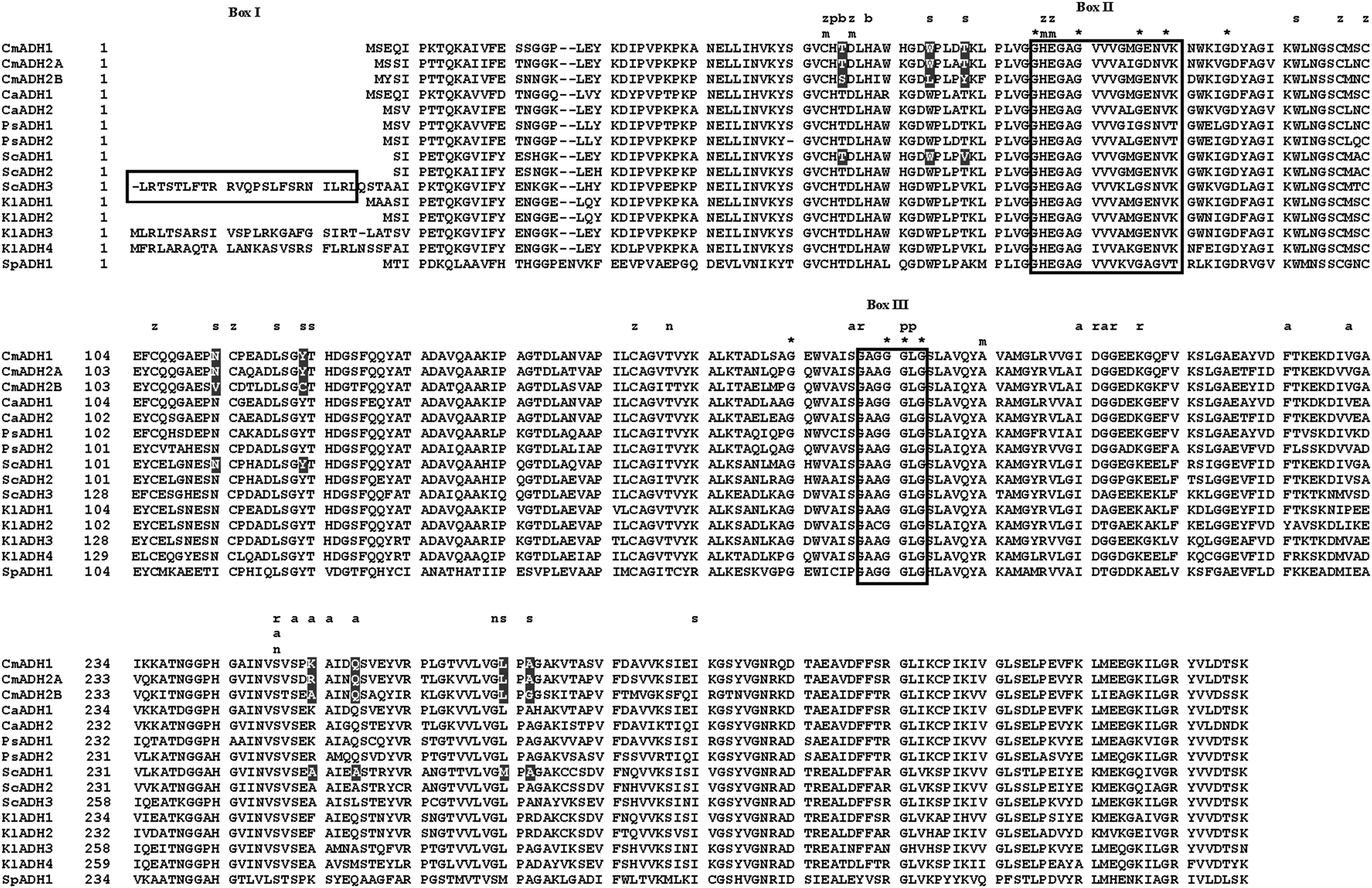

Supplement: Figure S2 — Alignment of the deduced amino acid sequences of CmADHs with those of other yeast ADHs. Numbering of amino acid corresponds to ScADH1. Residues that are involved in catalyses, are headed by letters: a, adenine binding pocket; r, adenosine ribose binding; p, pyrophosphate binding; n, binding of nicotinamide or nicotinamide ribose; s, substrate binding pocket; b, poton relay system; z, ligands of the active site zinc atom or ligands of the structural zinc atom. m, five more strictly conserved residues among the microbial ADHs. Asterisks indicate conserved amino acid residues, eight glycine residues and one valine of ADHs from divergent sources. Box I, mitochondrial targeting region; Box II, zinc-binding consensus; Box III, NAD(P)-binding motif. Reversed letters indicate the residues involved in cofactor binding, substrate binding or catalysis which are not conserved in three CmADHs and ScADH1. Accession numbers (from CaADH1 to SpADH1): X81694, XM_712556, AF008245, AF008244, V01292, Z49212, AY692988, XM_456023, X64397, X62766, X62767, AL032681. (2.49 MB TIF) [file pone.0011752.s004.tif]

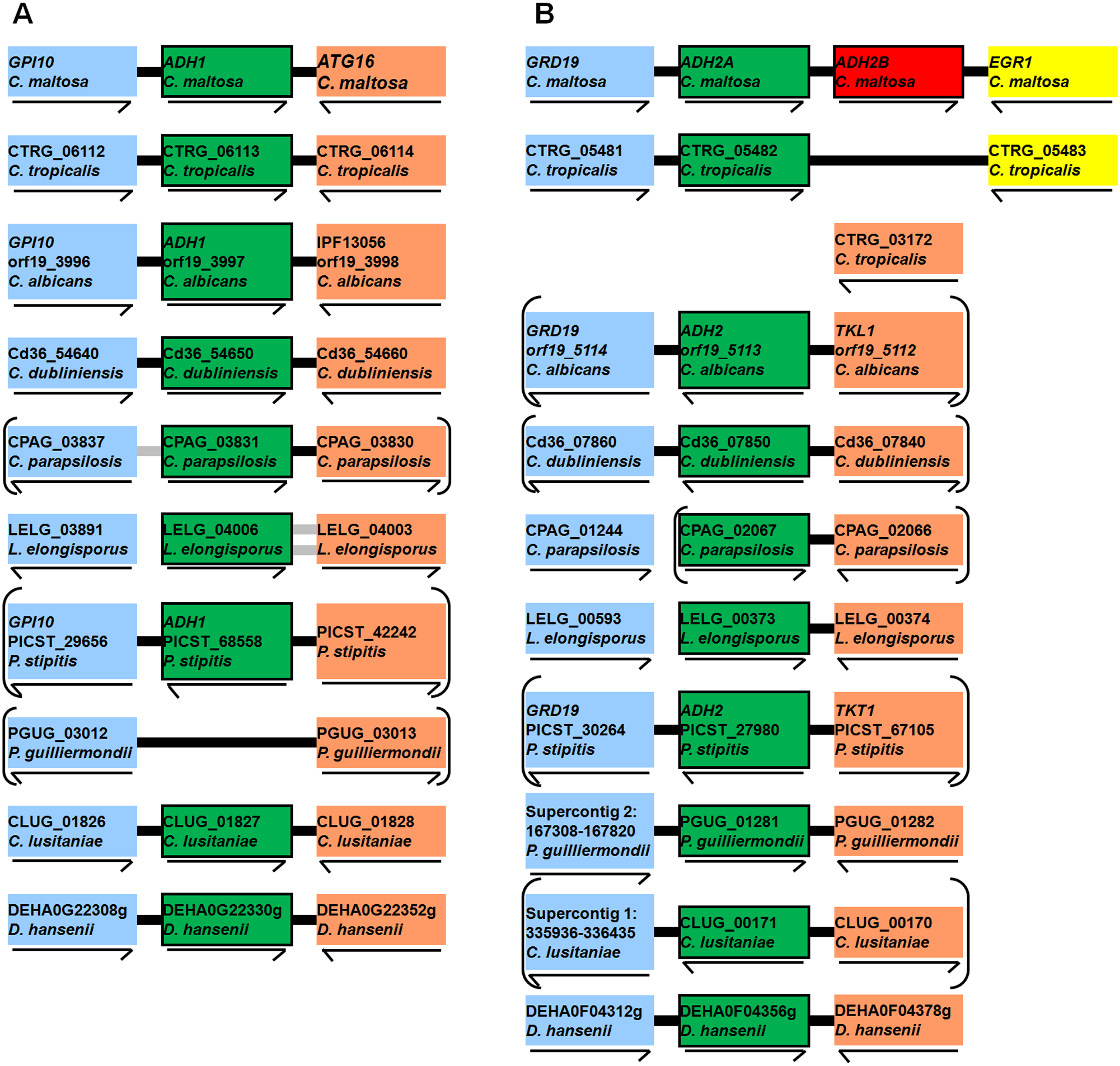

Supplement: Figure S3 — Genomic contexts of ADH1 (A) or ADH2 (B) homologs from species of the CTG clade are drawn according to their genomic sequences. Species names and gene identifiers are shown in each box. Orthologous gene boxes are represented in the same color. Arrows indicate directions of gene transcription and are not to scale. Connectors join nearby genes: a solid bar for adjacent genes, two gray bars for loci less than five genes apart and one gray bar for loci <20 genes apart. Genomic contexts in square brackets are conversed compared with C. maltosa. The species of the CTG clade exhibited highly conserved gene order around ADH1. In case of ADH2 region, C. maltosa had the same genomic context as C. tropicalis except that another ADH existed in C. maltosa, and the other species of the CTG clade had transketolase gene TKL1 instead of squalene epoxidase gene ERG1. In general, two ADH genes, ADH1 and ADH2, existed in all the species of the CTG clade except for Pichia (Candida) guilliermondii in this study, although the ADH loci were dispersed in Candida parapsilosis and Lodderomyces elongisporus. (1.34 MB TIF) [file pone.0011752.s005.tif]

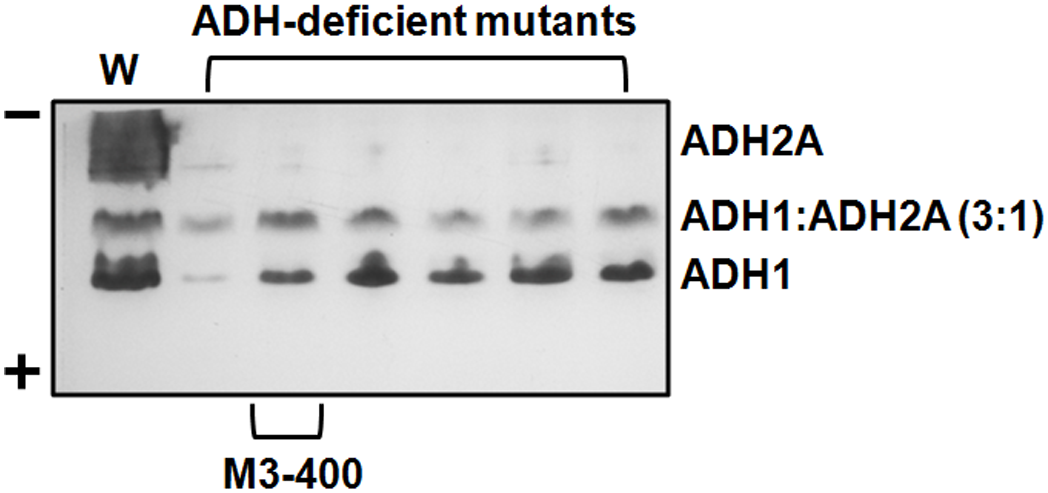

Supplement: Figure S4 — Characterization of ADH-deficient strain of C. maltosa by zymogram analysis. Cells were grown in YP medium containing 20 g/l glucose and harvested in the mid-exponential phase for zymogram analysis. Lane 1, wild-type strain Xu316. Lane 2–7, ADH-deficient mutants M3-360, M3-400, M1-400, M11-400, M12-400 and M15-400. (0.22 MB TIF) [file pone.0011752.s006.tif]
